# Oral ulcerations in an immunosuppressed pediatric patient

**DOI:** 10.1016/j.jdcr.2023.09.020

**Published:** 2023-10-01

**Authors:** Ansley DeVore, Margaret S. Newsome, Loretta S. Davis

**Affiliations:** a Department of Medicine, Medical University of South Carolina, Charleston, South Carolina; b Department of Dermatology, Medical College of Georgia, Augusta, Georgia

**Keywords:** aphthous ulcers, mTOR inhibitor-associated stomatitis, mucositis, oral ulcerations, sirolimus, stomatitis

## Case

**Fig 1:**
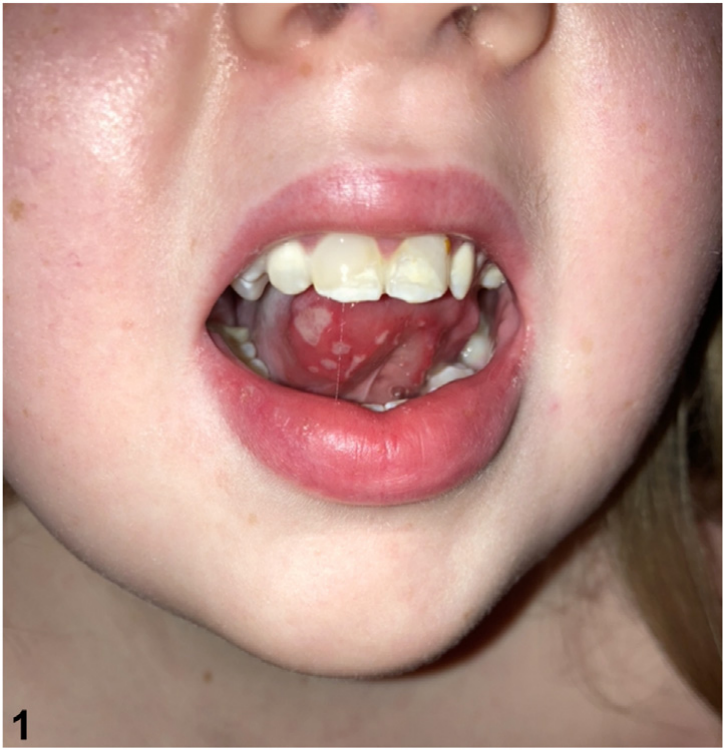

An 11-year-old girl with severe combined immunodeficiency, status postallogenic bone marrow transplantation 10 years prior, presented for painful mouth ulcers which developed shortly after tapering off chronic prednisone therapy. Ulcerations waxed and waned over 4 months, resulting in decreased oral intake. Review of systems was negative for fevers, arthralgias, rashes, gastrointestinal symptoms, and genital ulcerations. Medications included lisinopril, sirolimus, and intravenous infusions of daratumumab and abatacept. Her hematologist held her infusions without improvement. Examination revealed focal ulcerations on the ventral tongue (Fig 1). Review of photographs provided by her mother showed similar ulcerations on the inferior labial mucosa.

**Question 1: What is the likely etiology of these lesions?**

A. Herpes simplex virus
B. Recurrent aphthous ulceration
C. Chronic graft versus host disease (cGVHD)
D. Medication side effect
E. Severe combined immunodeficiency

**Answers:**

A. Herpes simplex virus – Incorrect. Herpes simplex virus oral ulcerations may occur in immunosuppressed patients typically presenting with grouped vesicles and erosions with irregular borders overlying gingival bone, hard palate, and vermilion lip. However, this patient has discrete tongue ulcerations with a clean fibrinous base.
B. Recurrent aphthous ulceration – Incorrect. Clinically similar in appearance, recurrent aphthae are a diagnosis of exclusion and not the best answer in this patient.
C. Chronic graft versus host disease (cGVHD) – Incorrect. Oral findings of cGVHD include lichen planus-like changes, salivary gland dysfunction, and microstomia due to tissue sclerosis. Over 90% of cGVHD occurs within the first year following transplantation.^1^ Ulcerations on a background of normal mucosa developing 3 years post-transplantation are not consistent with cGVHD.
D. Medication side effect – Correct. mTOR inhibitor-associated stomatitis (mIAS) is a well-documented side effect of mTOR inhibitors (eg, sirolimus) resembling common aphthae with an incidence ranging from 2% to 78%.^2^^,^^3^ Although mIAS pathogenesis is not fully understood, evidence suggests that oral microbiota changes may be contributory.^2^ non-steroidal anti-inflammatory drugs, trimethoprim-sulfamethoxazole, mycophenolate mofetil, and losartan, which were not taken by our patient, are also reported triggers of mucosal ulcerations. Angiotensin converting enzyme inhibitors are more likely to trigger oral lichenoid reactions rather than aphthous-like ulcers.
E. Severe combined immunodeficiency – Incorrect. Severe combined immunodeficiency encompasses a heterogeneous group of immunodeficiencies resulting from severe T and B cell defects. Mucosal candidiasis is the classic oral manifestation. Oral ulcerations are not typical.

**Question 2: What is the best initial management of the patient?**

A. Topical corticosteroids and hematology consultation regarding sirolimus usage
B. Topical analgesics while maintaining sirolimus dose
C. “Miracle Mouthwash” (ie, lidocaine, diphenhydramine, antacid) and stop sirolimus
D. Biopsy
E. Observation

**Answers:**

A. Topical corticosteroids and hematology consultation regarding sirolimus usage – Correct. Topical corticosteroids provide relief of mIAS, specifically reducing associated pain and lesion duration.^2^^,^^3^ However, the causative agent of the oral ulcerations must also be addressed. Since post-transplant immunosuppressive therapy is still required, decreasing sirolimus dose or switching to a different GVHD prophylactic agent is most appropriate. Our patient’s oral ulcerations resolved following sirolimus discontinuation recommended by her hematologist.
B. Topical analgesics while maintaining sirolimus dose – Incorrect. Analgesics may be useful adjuvants in some cases; however, analgesics do not address the underlying etiology of the mucosal lesions in this patient.
C. “Miracle Mouthwash” (ie, lidocaine, diphenhydramine, antacid) and stop sirolimus – Incorrect. This solution has been shown to be ineffective in improving the frequency or severity of sirolimus ulcerations as it provides only brief symptomatic relief.^3^ Additionally, it would be inappropriate to discontinue transplant GVHD prophylaxis without consulting the prescribing hematologist.
D. Biopsy – Incorrect. mIAS is a clinical diagnosis. Biopsy is not warranted, and there is a paucity of literature regarding specific histopathologic features of mIAS. One in vitro study demonstrated increased epithelial surface disruption, vacuole formation, and early dysplastic changes when oral mucosa was incubated in everolimus compared to control media.^4^
E. Observation – Incorrect. The patient has pain affecting oral intake and quality of life; therefore, intervention is necessary. Use of topical corticosteroids and management of sirolimus therapy address both symptoms and etiology of the lesions.^2^^,^^3^ On literature review, permanent resolution of mIAS does not occur while the inciting dose of sirolimus is continued.^3^

**Question 3: What is the patient’s most likely risk factor for developing this adverse event?**

A. Introduction of foreign lymphocytes
B. Hormonal alteration
C. Latent viral infection
D. Nutritional deficiency
E. Steroid discontinuation

**Answers:**

A. Introduction of foreign lymphocytes – Incorrect. Oral cGVHD, which can be the initial or only manifestation of cGVHD, is characterized by lichenoid mucositis. Although the pathogenesis is incompletely understood, activated alloreactive T-cells and other immune mediators from transplanted tissue are thought to drive the disease process. cGVHD does not typically present as discrete, aphthous-like lesions.^1^
B. Hormonal alteration – Incorrect. Hormonal changes were previously suggested as a cause of aphthous ulcers, but this theory has faded in popularity, lacking supporting evidence.^2^ Additionally, this patient is prepubertal.
C. Latent viral infection – Incorrect. Specimens of mIAS fail to demonstrate viral cytopathic changes, and patients do not typically respond to antivirals.^3^ As previously described, this patient did not have any of the specific features that typically characterize herpes simplex virus-associated oral ulcerations.
D. Nutritional deficiency – Incorrect. Nutritional deficiencies, such as inadequate iron or vitamins (B3, B9, B12, or C), may lead to oral ulcerations. This is a reasonable consideration in our patient with decreased oral intake; however, the onset of lesions preceded the decreased oral intake. Likewise, an association between nutritional deficiency and mIAS has not been reported.^2^
E. Steroid discontinuation – Correct. The ulcers occurred shortly after discontinuation of systemic corticosteroid therapy, suggesting that corticosteroid effects were preventing the onset of stomatitis. Indeed, a randomized control trial of patients with autosomal dominant polycystic kidney disease on sirolimus therapy reported decreased risk of mIAS with concomitant corticosteroid use.^5^

## Conflicts of interest

None disclosed.
